# Inter-clinician delineation variation for a new highly-conformal flank target volume in children with renal tumors: A SIOP-Renal Tumor Study Group international multicenter exercise

**DOI:** 10.1016/j.ctro.2021.03.001

**Published:** 2021-03-11

**Authors:** Joeri Mul, Patrick Melchior, Enrica Seravalli, Daniel Saunders, Stephanie Bolle, Alison L. Cameron, Kristin Gurtner, Semi Harrabi, Yasmin Lassen-Ramshad, Naomi Lavan, Henriette Magelssen, Henry Mandeville, Tom Boterberg, Petra S. Kroon, Alexis N.T.J. Kotte, Bianca A.W. Hoeben, Peter S.N. van Rossum, Martine van Grotel, Norbert Graf, Marry M. van den Heuvel-Eibrink, Christian Rübe, Geert O. Janssens

**Affiliations:** a Princess Máxima Center for Pediatric Oncology, Utrecht, the Netherlands; b Department of Radiation Oncology, University Medical Center Utrecht, Utrecht, the Netherlands; c Dept. of Radiation Oncology, Saarland University Hospital, Homburg, Germany; d Dept. of Clinical Oncology, The Christie Hospital, Manchester, United Kingdom; e Dept. of Radiation Oncology, Gustave Roussy, Villejuif, France; f Bristol Cancer Institute, University Hospitals, Bristol, United Kingdom; g Dept. of Radiation Oncology, University Hospital Carl Gustav Carus, Dresden, Germany; h Dept. of Radiation Oncology, Heidelberg University Hospital, Heidelberg, Germany; i Dept. of Clinical Oncology, Aarhus University Hospital, Aarhus, Denmark; j St. Luke’s Radiation Oncology Network, Dublin, Ireland; k Dept. of Oncology, Oslo University Hospital, Oslo, Norway; l Dept. of Clinical Oncology, The Royal Marsden NHS Foundation Trust, Sutton, United Kingdom; m Dept. of Radiation Oncology, Ghent University Hospital, Ghent, Belgium; n Dept. of Pediatric Oncology, Saarland University Hospital, Homburg, Germany

**Keywords:** Flank target volume, Highly-conformal radiotherapy, Inter-clinician variation, Pediatric renal tumors, Wilms tumor, Quality assurance, RTSG, Renal Tumor Study Group, SIOP, International Society for Pediatric Oncology, AP/PA, Anterior-Posterior/Posterior-Anterior, IGRT, Image-Guided Radiotherapy, OAR, organs at risk, RT, radiotherapy, MRI, Magnetic Resonance Imaging, CT, Computed Tomography, DICOM, Digital Imaging and Communications in Medicine, GTV_pre/post_, pre- and postoperative Gross Tumor Volume respectively, CTV-T, Clinical Target Volume of the primary Tumor, CTV-N, Clinical Target Volume of the lymph node area, DSC, Dice Similarity Coefficient, TV_ref_, reference target volumes, TV_intersect_, intersect target volume, ref, reference, part, participant, IMRT, Intensity-Modulated Radiotherapy, WT, Wilms’ tumor, IR, Intermediate-Risk, HR, High-Risk, n.a., not applicable, AA, abdominal aorta, IVC, inferior vena cava, RTOG, Radiation Oncology Group

## Abstract

**Background and purpose:**

Recently, the SIOP-RTSG developed a highly-conformal flank target volume definition for children with renal tumors. The aims of this study were to evaluate the inter-clinician delineation variation of this new target volume definition in an international multicenter setting and to explore the necessity of quality assurance.

**Materials and methods:**

Six pediatric renal cancer cases were transferred to ten radiation oncologists from seven European countries (‘participants’). These participants delineated the pre- and postoperative Gross Tumor Volume (GTV_pre/post_), and Clinical Target Volume (CTV) during two test phases (case 1–2 and 3–4), followed by guideline refinement and a quality assurance phase (case 5–6). Reference target volumes (TV_ref_) were established by three experienced radiation oncologists. The Dice Similarity Coefficient between the reference and participants (DSC_ref/part_) was calculated per case. Delineations of case 5–6 were graded by four independent reviewers as ‘per protocol’ (0–4 mm), ‘minor deviation’ (5–9 mm) or ‘major deviation’ (≥10 mm) from the delineation guideline using 18 standardized criteria. Also, a major deviation resulting in underestimation of the CTV_ref_ was regarded as an unacceptable variation.

**Results:**

A total of 57/60 delineation sets were completed. The median DSC_ref/part_ for the CTV was 0.55 without improvement after sequential cases (case 3–4 vs. case 5–6: p = 0.15). For case 5–6, a major deviation was found for 5/18, 12/17, 18/18 and 4/9 collected delineations of the GTV_pre_, GTV_post_, CTV-T and CTV-N, respectively. An unacceptable variation from the CTV_ref_ was found for 7/9 participants for case 5 and 6/9 participants for case 6.

**Conclusion:**

This international multicenter delineation exercise demonstrates that the new consensus for highly-conformal postoperative flank target volume delineation leads to geometrical variation among participants. Moreover, standardized review showed an unacceptable delineation variation in the majority of the participants. These findings strongly suggest the need for additional training and centralized pre-treatment review when this target volume delineation approach is implemented on a larger scale.

## Highlights

• Recently, highly-conformal target volumes for flank delineation were defined.
• Delineation variation of this target volume was assessed in an international setting.
• Ten radiation oncologist delineated the GTV and CTV of six individual cases.
• ‘Unacceptable’ delineation variation was found in a large number of participants.
• This indicates the need for central target volume review before radiotherapy onset.

## 1 Introduction

Most children with renal tumors who are treated according to the Renal Tumor Study Group (RTSG) protocols of the International Society for Pediatric Oncology (SIOP) receive upfront chemotherapy followed by nephrectomy. Data from the recent SIOP-2001 trial shows that 20–25% of these patients require postoperative flank irradiation as part of their first line treatment [1], [2]. For flank irradiation, two conventional opposing Anterior-Posterior/Posterior-Anterior (AP/PA) photon beams have been considered gold standard since the SIOP-1 trial (1971–1974) [3]. However, renal tumors arise from the retroperitoneal area and displace the organs anterior to the tumor. When performing surgery, the tumor is removed with limited risk of (intraperitoneal) tumor spill or macroscopic residual disease and surrounding organs shift into the surgical cavity [4]. Consequently, the volume irradiated by AP/PA photon beams includes a large amount of normal tissue.

Nowadays, advanced Image-Guided Radiotherapy (IGRT) techniques allow us to treat complex target volumes with high conformity. To exploit these favorable dose-volume characteristics, radiation oncologists affiliated with the SIOP-RTSG developed a consensus statement on highly-conformal flank target volume delineation for pediatric renal tumors [5]. As a result, the risk of inter-clinician variation is more substantial: underestimation of the target volume has the risk to increase locoregional failures, whereas overestimation of the target volume will limit the ability of modern IGRT techniques to spare healthy tissue. To assess the locoregional control of new flank target volumes combined with highly-conformal radiotherapy (RT) techniques, the SIOP-RTSG has the intention to launch a prospective multicenter study [5]. It is expected that during this study, prospective RT quality assurance by centralized review of target volumes and dosimetry will be compulsory to tackle the issue of inter-clinician variation, given earlier experiences with conventional flank delineation and in line with other recently launched pediatric cancer trials [6], [7], [8]. However, the estimated inter-clinician delineation variation and, subsequently, the need for centralized review of the new flank target volume has not been determined. Therefore, the development of the consensus on highly-conformal flank delineation was accompanied by a multicenter delineation exercise, during which the consensus guideline was continuously optimized based on the experiences of each delineation phase.

The aim of this study was to evaluate the inter-clinician variation of the new highly-conformal flank target volume delineation approach in an international multicenter setting using geometrical analyses and reviewing criteria in order to explore the necessity of centralized pre-treatment quality assurance.

## 2 Materials and methods

This exercise was reported according to the Guidelines for Reporting Reliability and Agreement Studies [9].

### 2.1 Patient selection

Six unique cases with a pediatric renal tumor eligible for flank irradiation based on the criteria defined in the SIOP-RTSG UMBRELLA 2016 protocol were selected for this delineation exercise (institutional review board approval number: 17-729/C) [1]. For each case, after preoperative chemotherapy, T1-weighted Magnetic Resonance Imaging (MRI) scans (Achieva 1.5T, Philips Medical Systems, Best, The Netherlands; slice thickness: 1.5 mm) with and without gadolinium contrast agent were acquired together with a postoperative planning Computed Tomography (CT) scans in RT treatment position (Brilliance, Philips Medical Systems, Best, The Netherlands, slice thickness of 2.0 mm). Essential clinical data to determine the extent of the area at risk were extracted from the radiology, surgery and pathology reports (Supplementary Table 1). Clinical data and imaging in Digital Imaging and Communications in Medicine (DICOM) format were anonymized and transferred from the coordinating center (University Medical Center Utrecht) to the participating centers using encrypted data exchange.

### 2.2 Procedure

#### 2.2.1 Preparation phase

**Fig. 1:**
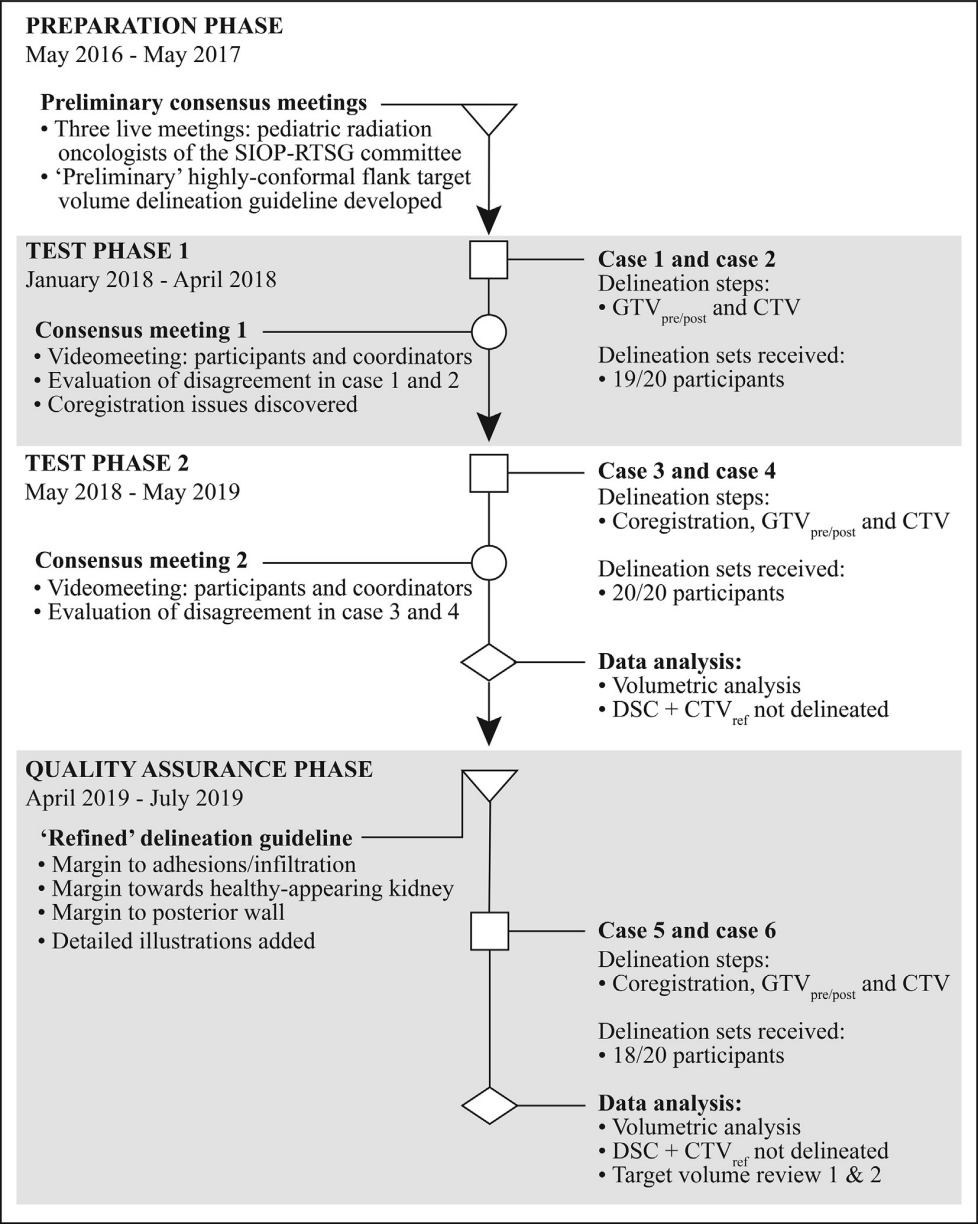
Flowchart depicting the procedure of the delineation exercise. *Abbreviations:* SIOP-RTSG, International Society for Pediatric Oncology – Renal Tumor Study Group; GTV_pre/post_, pre- and postoperative Gross Tumor Volume, respectively; CTV, Clinical Target Volume; DSC, Dice Similarity Coefficient.

Between May 2016 and May 2017, expert pediatric radiation oncologists of the SIOP-RTSG board (‘coordinators’) translated the conventional flank target volumes described in the ongoing UMBRELLA SIOP-RTSG-2016 protocol into a ‘preliminary’ highly-conformal flank delineation guideline during three live meetings [1]. Afterwards, radiation oncologists (‘participants’) from ten different centers in seven countries across Europe were invited to participate in a delineation exercise. Participants were asked to delineate the pre- and postoperative Gross Tumor Volume (GTV_pre/post_), as well as the Clinical Target Volume of the tumor bed and involved lymph node area when indicated (CTV-T and CTV-N, respectively) for all preselected cases using treatment contouring systems available at their institute. For each case, the contralateral kidney, spleen, liver, heart, lungs and vertebrae were delineated by a coordinating pediatric radiation oncologist (GJ) in order to reduce the total delineation time for the participants. The pancreas and intestine were delineated by the participants, since it is closely related to the construction of the target volumes. The delineation exercise was divided into three phases: two *test phases* and a *quality assurance phase* (Fig. 1).

#### 2.2.2 Test phases

During the *first test phase* (January 2018–April 2018), participants delineated the target volumes of case 1 and 2. The preoperative and postoperative scans of these cases had been co-registered in advance at the coordinating center. However, after all delineations were collected by the coordinating center, it was revealed that the rigid co-registration had been overruled by the delineation software at the participants’ departments. For this reason, detailed instructions on co-registration were amended to the ‘preliminary’ delineation guideline. Hence, in the *second test phase* (May 2018–May 2019), participants performed the co-registration themselves and delineated the target volumes of case 3 and 4. At the end of each test phase, a video meeting was organized between participants and coordinators to discuss inconsistencies between participants and to evaluate the need for refinement of the ‘preliminary’ delineation guideline.

#### 2.2.3 Quality assurance phase

At the beginning of the *quality assurance phase* (April 2019–July 2019), the ‘preliminary’ delineation guideline was refined by adding new recommendations and detailed illustrations of the delineation approach (Supplementary Table 2) [5]. In this phase, participants performed co-registration and delineated case 5 and 6 using the refined delineation guideline. The purpose of the *quality assurance phase* was to determine the inter-clinician variation using a standardized procedure to review the target volumes in addition to the geometrical analysis of the volumes.

### 2.3 Analysis

#### 2.3.1 Geometrical data analysis

Data analysis was limited to cases 3–6 due to the co-registration mismatch in case 1 and 2. Before each phase, a reference target volume (TV_ref_), consisting of the GTV_pre_, GTV_post_ and CTV, was established for each case by one of the coordinators (GJ), and subsequently validated by the other coordinators (PM, CR). The TV_ref_ was based on the ‘preliminary’ delineation guideline for case 3 and 4 and on the ‘refined’ delineation guideline for case 5 and 6. Afterwards, the volume of contours, Dice Similarity Coefficient (DSC) and the percentage of the TV_ref_ not delineated by participants were calculated using an in-house developed software tool [10].

The volume of contours (in mL) were calculated per participant, per case and per target volume. The DSC was used to determine the variation between two volumes and calculated as the intersect target volume (TV_intersect_) times two, divided by the sum of the two target volumes (TV_1_, TV_2_). The DSC ranges from 0 (no overlap between volumes) to 1 (perfect agreement between volumes).

$$\mathrm{DSC}\;=\frac{\text{2}\times {\text{TV}}_{\text{intersect}}}{{\text{TV}}_{\text{1}}+{\text{TV}}_{\text{2}}}$$

DSCs were calculated in a pairwise fashion between each participant and the reference (DSC_ref/part_), as well as between the participants only (DSC_part/part_) for each target volume per case. The percentage of TV_ref_ not delineated by a participant was calculated for each target volume per case to reflect the amount of underestimated treatment volume. Zero percent indicated that no part of the TV_ref_ was included by the corresponding target volume of the participant, while 100% indicated that all of the TV_ref_ was delineated by the participant.

$$\mathrm{Reference}\;volume\;not\;delineated\;in\;\%\;=\frac{{\text{TV}}_{\text{ref}}-{\text{TV}}_{\text{intersect}}}{{\text{TV}}_{\text{ref}}\;}\times \text{100}$$

#### 2.3.2 Target volume review

Target volume review according to the ‘refined’ delineation guideline was performed for case 5 and 6 only using a maximum of 18 standardized criteria depending on the clinical situation. These criteria cover the five major steps in the delineation process: one for co-registration, one for GTV_pre_, seven for GTV_post_, six for CTV-T and three for CTV-N [5] (Supplementary Table 2).

For the first part of the review, delineations per case per participant were graded by two independent reviewers (BH, PvR) and one reviewer with prior involvement in the delineation exercise (JM). Since a deviation occurring in each delineation step may cause a systematic error in the succeeding steps, each delineation step was reviewed separately. Subsequently, every deviation was appointed to the violation of a specific criterion. Deviations from the criteria were measured in the axial view using a point-to-point distance tool and categorized as either per protocol (0–4 mm), minor deviation (5–9 mm) or major deviation (≥10 mm). Deviations were only graded as minor or major when present in 3 or more consecutive axial slices. Major deviations were subdivided into deviations leading to a potential underestimation or overestimation of the target volume. Discrepancies between reviewers were resolved collectively.

For the second part of the review, a reference pediatric radiation oncologist (GJ) and two independent reviewers (BH, PvR) graded deviations from the CTV_ref_ by each participant in six directions of the CTV (anterior, posterior, medial, lateral, cranial and caudal) using automated expansions of the CTV_ref_. A major deviation in one direction of the CTV resulting in underestimation with potential increased risk of locoregional failure was regarded as an unacceptable variation. All minor deviations and major deviations leading to an overestimation were considered acceptable.

### 2.4 Statistical analysis

The median of the volumes, the DSC_ref/part,_ the DSC_part/part_ and the TV_ref_ not delineated by participants were generated. The One-Sample Wilcoxon signed-rank test was used to test the difference between the size of the CTV_part_ and the CTV_ref_ for each case. The Wilcoxon signed-rank test was used to test whether a significant increase of the DSC_ref/part_ was obtained between the mean of case 3 and 4 (‘second test phase’) and the mean of case 5 and 6 (‘quality control phase’). The Related-Samples Friedman's Two-Way Analysis of Variance by Ranks with the Wilcoxon signed-rank test as post-hoc analysis was used to test the difference of CTV_ref_ not delineated by the participants between cases, and to test the difference between the mean DSC_ref/part_ of the GTV_pre_ versus the GTV_post_ versus the CTV of all of cases combined. Additionally, the difference between the DSC_ref/part_ and the DSC_part/part_ was tested using the Mann-Whitney *U* test. A p-value of <0.05 was chosen to indicate statistical significance. Data were analyzed using statistical software SPSS

## 3 Results

### 3.1 Data collection

At the end of the quality-control phase, a total of 57/60 delineation sets had been collected by the coordinating center. One participating center was unable to delineate case 1, 5 and 6 within the given timeframes.

### 3.2 Geometrical data analysis

Table 1 demonstrates the absolute volume of the GTV_pre_, GTV_post_ and CTV of each participant compared to the reference target volumes for case 3–6. For all cases, CTV_obs_ was not significantly different compared to the CTV_ref._ Considering each individual participant, the maximum difference in size of the CTV_part_ compared to the CTV_ref_ ranged from minus 68 mL to plus 234 mL.

**Table 1:** Volumetric analysis of case 3–6 for a total of 30/40 completed delineation sets by 10 participants.

		Reference volumes	Participants volumes		mean Δ(part – ref)	P-value*
	Target volume	mL	N	median mL (min–max)	mL (%)	
Case 3	GTV_pre_	39.4	9	38.6 (29.8–94.0)	3.8 (8.9)	
	GTV_post_	8.9	9	12.9 (8.4–78.5)	16.2 (64.6)	
	CTV	71.8	10	82.8 (62.3–262.5)	42.4 (37.1)	0.20
Case 4	GTV_pre_	299.9	10	271.0 (227.9–295.1)	−34.1 (−12.9)	
	GTV_post_	59.8	10	41.5 (24.1–111.7)	−12.5 (−26.5)	
	CTV	124.1	10	94.3 (56.4–231.2)	−1.6 (−1.3)	0.72
Case 5	GTV_pre_	133.2	8	147.2 (123.9–162.0)	13.2 (9.0)	
	GTV_post_	15.2	8	28.6 (12.0–54.1)	15.5 (50.5)	
	CTV	175.2	9	217.5 (149.9–409.7)	63.2 (26.5)	0.05
Case 6	GTV_pre_	164.7	9	163.3 (148.1–172.2)	−4.3 (−2.7)	
	GTV_post_	17.3	9	20.6 (10.6–42.1)	6.5 (27.6)	
	CTV	83.8	9	108.3 (49.2–193.7)	34.2 (29.0)	0.14

*For each case, the difference between the mean CTV_obs_ and the CTV_ref_ was tested using a One-Sample Wilcoxon Signed Rank test. A p-value of < 0.05 was chosen to indicate statistical significance.

**Fig. 2:**
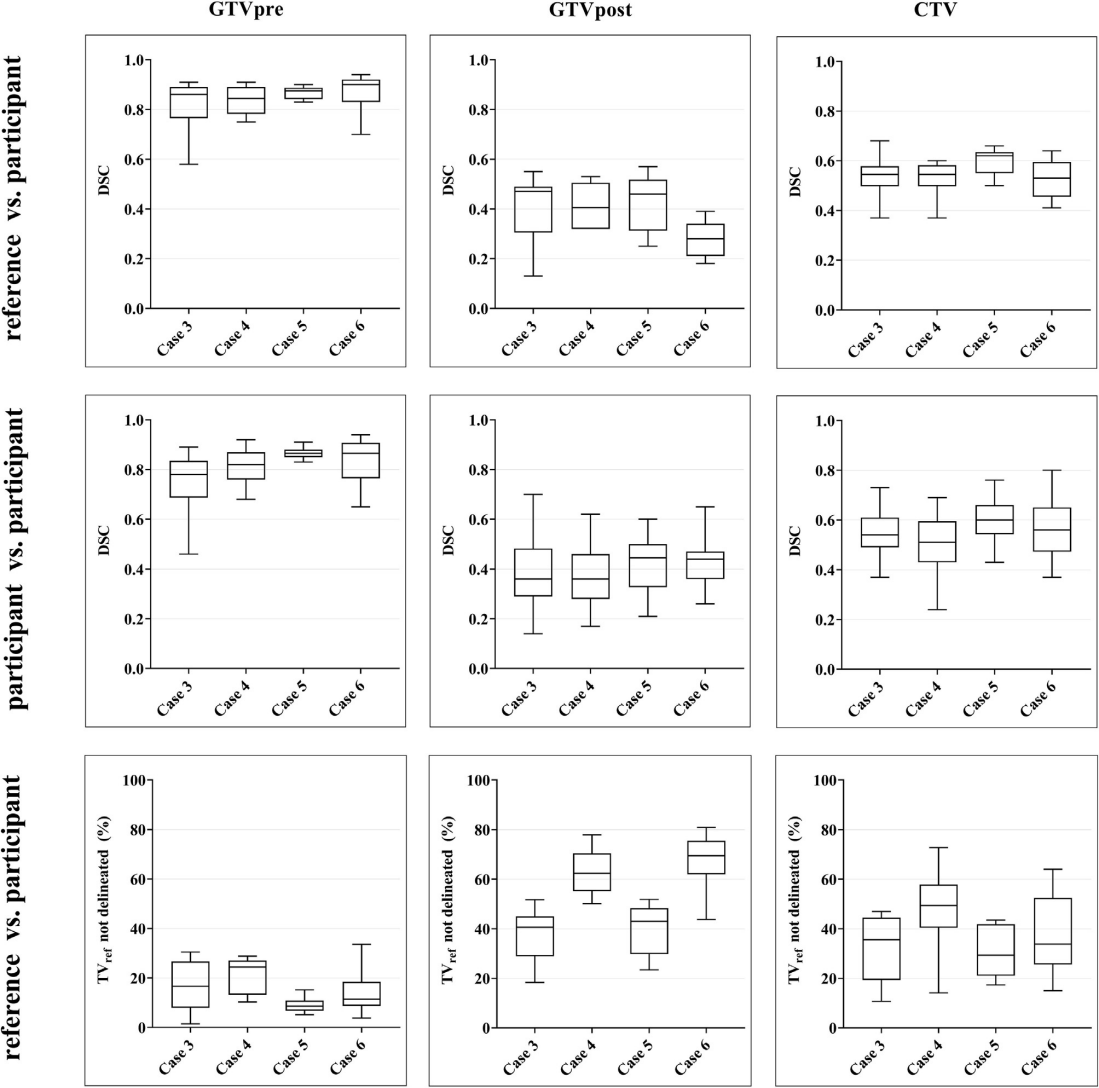
Boxplots for DSC_ref/obs_, DSC_obs/obs_ and TV_ref_ not delineated for case 3–6 per target volume. For a total of 38 delineation sets (2 missing) that were completed by the participants, the boxplots depict the DSC_ref/part_ (*upper row*), DSC_part/part_ (*middle row*) and TV_ref_ not delineated (*lower row*) of case 3–6 per target volume. *Abbreviations:* TV_ref_, reference target volume; DSC, Dice Similarity Coefficient; GTV_pre/post_, pre- and postoperative Gross Tumor Volume, respectively; CTV, Clinical Target Volume.

**Fig. 3:**
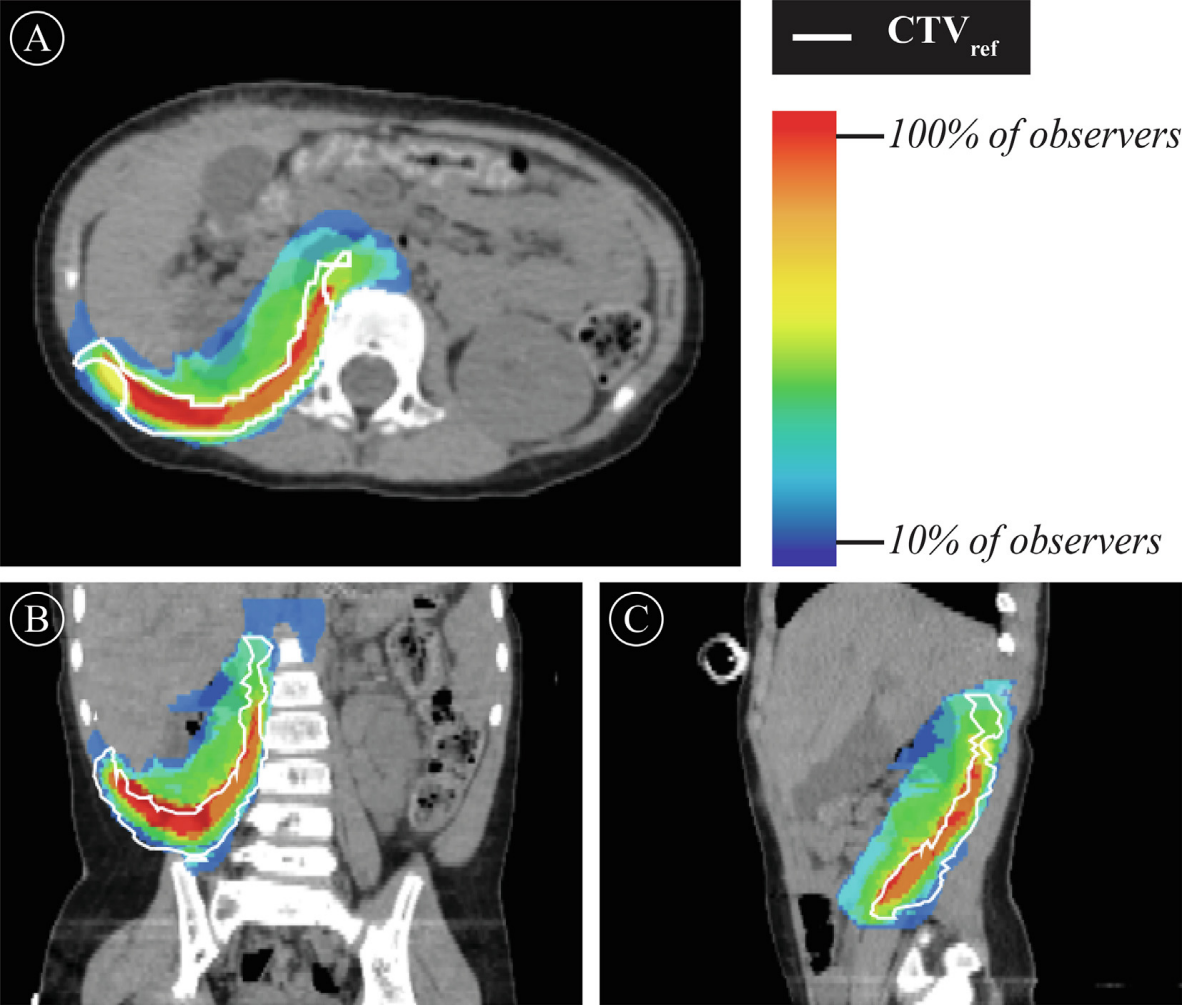
Count map of case 6 (female 2 years old, stage II WT-HR): the degree of agreement between the CTV_part_ (n = 9/10), alongside the CTV_ref_ (n = 1). For a total of 9/10 participants, overlap between the CTV_part_ for case 6 alongside the CTV_ref_ (*white*) is shown on the axial (3A), coronal (3B) and sagittal (3C) postoperative CT. Red and blue zones depict 100% and 10% agreement between participants only, respectively. *Abbreviations:* WT, Wilms’ tumor; HR, High-Risk; CTV, Clinical Target Volume. (For interpretation of the references to colour in this figure legend, the reader is referred to the web version of this article.)

The boxplots in Fig. 2 illustrate the variation in DSC between the reference and the participants (DSC_ref/part_), the variation between the participants only (DSC_part/part_) for each target volume per case and the percentage of TV_ref_ not delineated by a participant. All cases combined, the DSC_ref/part_ was better for the GTV_pre_ (median = 0.87) compared to the GTV_post_ (median = 0.39, p = 0.03) and CTV (median = 0.55, p = 0.02). No significant difference in DSC_ref/part_ for the CTV was observed between the ‘*test phase*’ and the ‘*quality assurance phase*’ (case 3/4 vs. case 5/6: p = 0.15, standard error = 8.43). For the CTV of each case, the DSC_ref/part_ and the DSC_part/part_ were not significantly different (case 3: p = 0.84; case 4: p = 0.59; case 5: p = 0.84; case 6: p = 0.32). The percentage of CTV_ref_ not delineated by the CTV of all participants for case 3–6 ranged between 11% and 73% (median = 35%) and did not significantly differ between cases (p = 0.17) (Fig. 3; Supplementary Table 3).

### 3.3 Target volume review

Firstly, case 5 and 6 were reviewed by grading each step in the delineation process separately. One or more major deviations were found in 2/18, 5/18, 12/17, 18/18 and 4/9 participants for co-registration, GTV_pre_, GTV_post_, CTV-T and CTV-N, respectively (Supplementary Table 4). The criteria with highest number of major deviations are CTV-T criterion 3 (‘healthy-appearing kidney’, n = 14/18), GTV_post_ criterion 3 (‘healthy-appearing kidney’, n = 8/17) and CTV-T criterion 2 (‘organs at risk’, n = 9/18) (Table 2; Supplementary Table 2). Twenty-nine of the 44 observed major deviations were the result of an overestimation, while 15 of the 44 observed major deviations were caused by an underestimation

**Table 2:** Review of case 5 and 6: total number of deviations per criterion.

		Per protocol	Minor	Major (under-/overestimation)
	Delineations received	0–4 mm	5–9 mm	≥10 mm
Coregistration				
Criterion 1: coregistration	18	15	1	2 (n.a./n.a.)

GTV_pre_				
Criterion 1: macroscopic tumor	18	11	2	5 (0/5)

GTV_post_				
Criterion 1: OARs	17	17	0	0 (n.a./0)
Criterion 2: contact zone of GTV_pre_	17	15	1	1 (0/1)
Criterion 3: healthy appearing kidney	17	8	1	8 (0/8)

CTV-T				
Criterion 1: isotropic margin	18	17	0	1 (0/1)
Criterion 2: OARs	18	8	1	9 (n.a./9)
Criterion 3: healthy appearing kidney	18	3	1	14 (13/1)
Criterion 4: posterior wall	18	16	0	2 (0/2)

CTV-N*				
Criterion 1: area around AA/IVC	9	4	2	3 (1/2)
Criterion 2: cranial border	9	7	0	2 (2/0)
Criterion 3: caudal border	9	7	2	0 (0/0)

*The delineation of a CTV-N was only indicated for case 5.

*Abbreviations:* n.a., not applicable; GTV_pre/post_, pre- and postoperative Gross Tumor Volume; OAR, organs at risk; CTV-T/N, Clinical Target Volume of the tumor/involved lymph node area; AA, abdominal aorta; IVC, inferior vena cava.

**Fig. 4:**
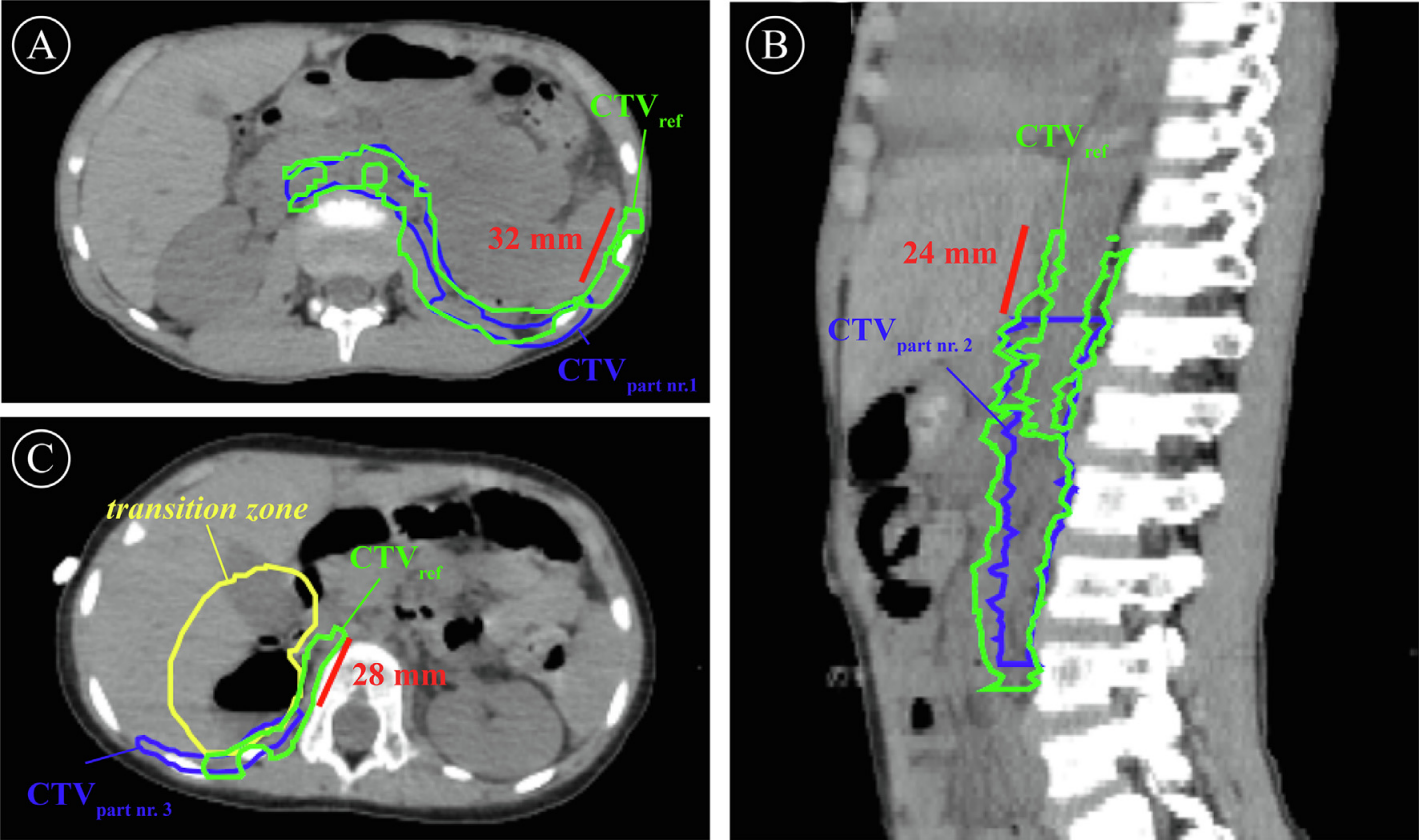
Three examples of an unacceptable variation by a participant observed during target volume review of case 5 and case 6. The postoperative CT’s show the CTV_ref_ (*green*) compared to the CTV of three different participants (*blue*) who were found to have an unacceptable variation measured with a point-to-point ruler (*red*). For case 5, participant number 1 underestimates the lateral margin of the CTV-T by 32 mm (4A, axial slice), and participant number 2 underestimates the cranial border of the CTV-N by 24 mm (4B, sagittal slice). For case 6, participant 3 underestimates the CTV-T at the medial side towards the transition zone (*yellow*) (4C, axial slice). (For interpretation of the references to colour in this figure legend, the reader is referred to the web version of this article.)

For the second part of the review, each CTV_obs_ was graded by the deviation from the CTV_ref_. An unacceptable variation from the CTV_ref_ was found in 7/9 participants for case 5 and 6/9 participants for case 6 (Fig. 4).

## 4 Discussion

In the current study, ten radiation oncologists from seven European countries delineated the pre- and postoperative GTV, as well as the CTV of six unique renal tumor cases in order to evaluate the inter-clinician variation of a new flank target volume delineation approach [5]. The median DSC was 0.55, expressing the overlap between the CTV of participants and the reference CTV, while the median underestimation of the reference CTV by the participants ranged between 29% and 47%. Additionally, standardized review of the delineations showed that an unacceptable underestimation of a reference CTV was present in 7/9 participants for case 5 and 6/9 participants for case 6.

Volume and measurements of overlap, like the DSC and Generalized conformity index, are commonly used metrics to determine inter-clinician delineation variation [11], [12], [13]. In a nationwide French study, the CTV agreement for conventional flank irradiation, as defined in the SIOP-2001 protocol, ranged from 0.50 to 0.64 between five RT teams [6]. In our study, the median DSC_ref/part_ for the CTV ranged from 0.53 to 0.62. Despite two consensus meetings with the participants and refinement of the preliminary delineation guideline, no significant improvement of inter-clinician variation was observed during this study. This lack of improvement might be caused by the complexity of postoperative tumor reconstruction, the diversity of clinical presentations and the rarity of pediatric (renal) tumors in general [14]. Moreover, the participants did not receive any training prior to this study and no feedback was provided regarding their individual performance during the study. This might have reduced the number of errors, as demonstrated in other clinical settings [15], [16], [17]. Finally, it is also important to consider that the DSC is generally more severely affected by variation in case of small, concave volumes like the postoperative tumor bed, compared to larger, spherical volumes like the GTV_pre_, as also demonstrated in this study [11], [18].

In order to evaluate the effect of variation on the potential clinical outcome of patients, a standardized review of all delineations was performed using objective criteria that reflect the recommendations from the refined delineation guideline [5]. When each step in the delineation process was graded independently during the first review, major deviations predominantly occurred for the margins towards the healthy-appearing kidney tissue, the removal of uninvolved OARs from the CTV-T, and the cranial margin of the CTV-N. This indicates where the delineation guideline could be improved or where additional attention during the reviewing process is appropriate.

The second review showed an unacceptable deviation from the CTV_ref_ (i.e. leading to significant underestimation) in the majority of the participants. In adult cancer, it is known that RT protocol violation may increase the risk of treatment failure [19], [20]. The Radiation Oncology Group (RTOG) revealed that failure to adhere to RT guidelines was associated with an increased risk of locoregional failure during a phase III study for pancreatic cancer, as well as during a multi-institutional trial for early-stage gastrointestinal cancer using Intensity-Modulated Radiotherapy (IMRT) [21], [22]. Also, a large phase III trial of advanced head and neck cancers using prospective quality assurance in 81 Australian centers found a statistically significant 2-year locoregional control rate of 54% versus 78% for patients with and without major deviations, respectively [23]. Less is known about the negative impact of RT protocol violation on treatment outcome for pediatric cancers. Carrie et al. reviewed the treatment plans of 174 medulloblastoma patients and demonstrated a strong correlation between the number of major target volume deviations and the risk of tumor relapse [24]. While the rate of protocol deviations found in our study is based upon a carefully established reference target volume, the true effect of underestimation can only be determined when comparing clinical outcome and target volume review. However, given the low numbers of locoregional failure for WT compared to medulloblastoma patients, it will be more challenging to demonstrate the impact of major deviations on outcome when this new RT techniques is introduced on a larger scale [2], [25], [26], [27], [28]. Overestimation of the target volume was not regarded as an unacceptable variation in our analysis. However, the degree of overestimation should also be evaluated within central target volume review in order to prevent unnecessary violation of normal tissue constraints like the spleen, tail of the pancreas or the heart. The design of this study was chosen to mimic daily clinical practice with cases representing a wide range of clinical situations. Also, ten radiation oncologists from seven different countries in Europe participated in this study, reflecting the inter-clinician variability in an international multicenter setting. Furthermore, this study implemented a review approach similar to modern quality assurance initiatives [7], [8]. However, the use of multiple review criteria and establishing reference target volumes might not be preferable for real-time pre-treatment quality assurance, since it is complex and time consuming for reviewers and RT for renal cancers has to start shortly after surgery [29]. Nevertheless, the criteria generated for this delineation exercise could be a good frame of reference since they reflect all recommendations from the consensus statement on the new flank target volume definition [5]. Since this study aimed to evaluate inter-clinician delineation variation only, dosimetric analyses were not included in this study, but are normally part of the RT quality assurance.

In conclusion, this international multicenter delineation exercise demonstrates that this new approach for flank target volume delineation leads to geometrical variation among clinicians. Standardized review using a reference CTV shows that major deviations leading to an underestimation of the reference CTV occurred in the majority of the participants. These findings strongly suggest the need for additional training and centralized pre-treatment review when this highly-conformal target volume delineation approach is implemented during a SIOP-RTSG endorsed prospective multicenter study.

## 5 Data availability statement

Research data are available upon request to the corresponding author.

## Funding source

KiKa (Children Cancer-free) Foundation, grant number 328.

## CRediT authorship contribution statement

**Joeri Mul:** Data curation, Formal analysis, Investigation, Methodology, Project administration, Visualization, Writing – original draft, Writing - review & editing. **Patrick Melchior:** Investigation, Methodology, Writing – original draft, Writing - review & editing. **Enrica Seravalli:** Project administration, Methodology, Writing - review & editing. **Daniel Saunders:** Investigation, Methodology, Writing - review & editing. **Stephanie Bolle:** Investigation, Methodology, Writing - review & editing. **Alison L. Cameron:** Investigation, Methodology, Writing - review & editing. **Kristin Gurtner:** Investigation, Methodology, Writing - review & editing. **Semi Harrabi:** Investigation, Methodology, Writing - review & editing. **Yasmin Lassen-Ramshad:** Investigation, Methodology, Writing - review & editing. **Naomi Lavan:** Investigation, Methodology, Writing - review & editing. **Tom Boterberg:** Investigation, Methodology, Writing - review & editing. **Henriette Magelssen:** Investigation, Methodology, Writing - review & editing. **Henry Mandeville:** Investigation, Methodology, Writing - review & editing. **Petra S. Kroon:** Data curation, Investigation, Writing - review & editing. **Alexis N.T.J. Kotte:** Data curation, Formal analysis, Investigation, Writing - review & editing. **Bianca Hoeben:** Investigation, Methodology, Writing - review & editing. **Peter S.N. van Rossum:** Investigation, Methodology, Writing - review & editing. **Martine van Grotel:** Investigation, Methodology, Supervision, Writing - review & editing. **Norbert Graf:** Investigation, Methodology, Writing - review & editing. **Marry M. van den Heuvel-Eibrink:** Conceptualization, Investigation, Methodology, Supervision, Writing - review & editing. **Christian Rübe:** Investigation, Methodology, Writing - review & editing. **Geert O. Janssens:** Conceptualization, Formal analysis, Funding acquisition, Investigation, Methodology, Supervision, Writing – original draft.

## Declaration of Competing Interest

The authors declare that they have no known competing financial interests or personal relationships that could have appeared to influence the work reported in this paper.
