# Supplementary material for: Inter-clinician delineation variation for a new highly-conformal flank target volume in children with renal tumors: A SIOP-Renal Tumor Study Group international multicenter exercise
Source: Clin Transl Radiat Oncol. 2021 Mar 11;28:39–47. doi: 10.1016/j.ctro.2021.03.001 (PMC7995478; doi:10.1016/j.ctro.2021.03.001)
Supplement: Supplementary data 1 [file mmc1.docx]

**Supplementary Table 1**

Detailed case characteristics

|  | **Gender (male/female)** | **Age (years)** | **Histological risk (IR/HR)** | **Lateralization (left/right)** | **Lymph node involvement (+/-)** | **Resection margin (+/-)** | **Arterial or venous involvement (+/-)** | **Adhesions (+/-)** | **Stage**  **(II-III)** |
| --- | --- | --- | --- | --- | --- | --- | --- | --- | --- |
| **Case 1** | female | 2 | IR | right | - | + | - | - | III |
| **Case 2** | male | 3 | IR | right | - | + | + (IVC) | - | III |
| **Case 3** | female | 3 | IR | left | + | + | - | - | III |
| **Case 4** | male | 2 | IR | right | - | + | + (renal vein) | + | III |
| **Case 5** | male | 9 | IR | left | + | - | - | - | III |
| **Case 6** | female | 2 | HR | right | - | - | - | - | II |
| **Abbreviations:** IR, Intermediate-Risk; HR, High-Risk; IVC, inferior vena cava. | | | | | | | | | |

**Supplementary Table 2**

Eighteen standardized reviewing criteria covering five major steps in the flank target volume delineation process

| **Step 1: image co-registration** | | **Per protocol** | **Minor deviation** | **Major deviation** | **Effect of deviation** |
| --- | --- | --- | --- | --- | --- |
| *Criterion 1:* | Co-registration of preoperative imaging with postoperative planning CT. | 0-5 mm | 6-9 mm | ≥10 mm | n.a. |
| **Step 2: GTV_pre_** | | **Per protocol** | **Minor deviation** | **Major deviation** | **Effect of deviation** |
| *Criterion 1:* | GTV_pre_ includes all of the macroscopic tissue of the primary tumor site after preoperative chemotherapy. | 0-5 mm | 6-9 mm | ≥10 mm | over- or underestimation |
| **Step 3A: GTV_post_ (common cases)** | | **Per protocol** | **Minor deviation** | **Major deviation** | **Effect of deviation** |
| *Criterion 1:* | All uninvolved OAR are excluded from the GTV-T_post_. | 0-5 mm | 6-9 mm | ≥10 mm | overestimation |
| *Criterion 2:* | In case of a posterior tumor, the GTV-T_post_ is defined by the *entire contact zone* of the GTV-T_pre_ with the abdominal wall. | 0-5 mm | 6-9 mm | ≥10 mm | over- or underestimation |
| *Criterion 3:* | In case of an anterior tumor, the GTV-T_post_ is defined by the contact zone of the GTV-T_pre_ with the abdominal wall, while the lateral clip/fictional line determines the lateral margin. | 0-5 mm | 6-9 mm | ≥10 mm | over- or underestimation |
| *Criterion 4:* | The GTV-T_post_ does not include contact zone of the healthy-appearing kidney. | 0-5 mm | 6-9 mm | ≥10 mm | over- or underestimation |
| **Step 3B: GTV_post_ (uncommon cases)** | | **Per protocol** | **Minor deviation** | **Major deviation** | **Effect of deviation** |
| *Criterion 5:* | In case of an inferior vena cava thrombus, all affected parts of the caval and renal veins are included. | 0-5 mm | 6-9 mm | ≥10 mm | over- or underestimation |
| *Criterion 6:* | In case of adhesions to or infiltration of an OAR, the whole contact zone of the GTV-T_pre_ with the involved OAR is included. | 0-5 mm | 6-9 mm | ≥10 mm | over- or underestimation |
| *Criterion 7:* | In case of *multifocal disease*, only the tumor responsible for indicating flank irradiation is included in the GTV-T_post_. | 0-5 mm | 6-9 mm | ≥10 mm | over- or underestimation |
| **Step 4A: CTV-T (common cases)** | | **Per protocol** | **Minor deviation** | **Major deviation** | **Effect of deviation** |
| *Criterion 1:* | A isotropic margin of 10 mm is added to the GTV-T_post_ | 0-5 mm | 6-9 mm | ≥10 mm | over- or underestimation |
| *Criterion 2:* | All uninvolved OAR are excluded from the CTV-T. | 0-5 mm | 6-9 mm | ≥10 mm | overestimation |
| *Criterion 3:* | The CTV-T margin is increased to 20 mm along the posterior border of the healthy-appearing ipsilateral kidney. | 0-5 mm | 6-9 mm | ≥10 mm | over- or underestimation |
| *Criterion 4:* | Expansion of the CTV-T into the posterior wall is limited to 5 mm instead of 10 mm. | 0-5 mm | 6-9 mm | ≥10 mm | over- or underestimation |
| **Step 4B: CTV-T (uncommon cases)** | |  |  |  |  |
| *Criterion 5:* | In case of adhesions to or infiltration of an OAR, a CTV margin of 5 or 10 mm into the OAR is added, respectively. | 0-5 mm | 6-9 mm | ≥10 mm | over- or underestimation |
| *Criterion 6:* | For patients with tumor extension into the vena cava inferior, a 10 mm cranio-caudal CTV-T expansion around the thrombus is added, limited by the vessel wall. | 0-5 mm | 6-9 mm | ≥10 mm | over- or underestimation |
| **Step 5: CTV-N** | | **Per protocol** | **Minor deviation** | **Major deviation** | **Effect of deviation** |
| *Criterion 1:* | The CTV-N includes the lymph node area around the ipsilateral renal vessels and the lymph nodes entangled around the aorta and vena cava. | 0-5 mm | 6-9 mm | ≥10 mm | over- or underestimation |
| *Criterion 2:* | The cranial border of the CTV-N is placed at the level of T10/11. | 0-5 mm | 6-9 mm | ≥10 mm | over- or underestimation |
| *Criterion 3:* | The caudal border of the CTV-N is placed at the bifurcation of the aorta. | 0-5 mm | 6-9 mm | ≥10 mm | over- or underestimation |
| *Indicates the recommendations that were added to the preliminary delineation guideline after refinement.  **Abbreviations:** mm, millimeter; GTV_pre/post_, pre- and postoperative Gross Tumor Volume; OAR, organs at risk; CTV-T/N, Clinical Target Volume of the tumor/involved lymph node area. | | | | | |

**Supplementary Table 3**

Measurements of overlap between the reference and participants (ref/part), as well as between participants only (part/part).

|  | **Case 3** | **Case 4** | **Case 5** | **Case 6** |
| --- | --- | --- | --- | --- |
| **DSC_ref/part_** |  |  |  |  |
| median | 0.55 | 0.55 | 0.62 | 0.53 |
| min - max | 0.37 - 0.68 | 0.37 - 0.60 | 0.50 – 0.66 | 0.41 – 0.64 |
| **DSC_part/part_** |  |  |  |  |
| median | 0.39 | 0.51 | 0.60 | 0.56 |
| min - max | 0.37 – 0.73 | 0.24 – 0.69 | 0.43 – 0.76 | 0.37 – 0.80 |
| **CTV_ref_ not delineated by CTV_part_ (%)** |  |  |  |  |
| median | 35.6 | 47.1 | 29.3 | 33.8 |
| min - max | 10.7 – 47.0 | 14.2 – 72.8 | 17.3 – 43.5 | 15.0 – 64.0 |
| **Abbreviations:** DSC, Dice Similarity Coefficient; CTV, Clinical Target Volume. | | | | |

**Supplementary table 4**

Review of case 5 and 6: highest deviation per delineation step

| **Target volume** | **Delineations received** | **Per protocol** | **Minor** | **Major** |
| --- | --- | --- | --- | --- |
| *co-registration* | 18 | 15 | 1 | 2 |
| *GTV_pre_* | 18 | 11 | 2 | 5 |
| *GTV_post_* | 17 | 3 | 2 | 12 |
| *CTV-T* | 18 | 0 | 0 | 18 |
| *CTV-N** | 9 | 2 | 3 | 4 |
| *The delineation of a CTV-N was only indicated for case 5.  **Abbreviations:** GTV_pre/post_, Gross Tumor Volume of the primary tumor before and after surgery; CTV, Clinical Target Volume. | | | | |
